# Physical Exams and Healthcare Utilization and Expenditures Among Mid-Aged and Older Adults in China

**DOI:** 10.1093/geroni/igaf122.261

**Published:** 2025-12-31

**Authors:** Sijiu Wang, Tianzi Li

**Affiliations:** Tsinghua University, Beijing, Beijing, China (People’s Republic); Tsinghua University, Beijing, Beijing, China (People’s Republic)

## Abstract

As populations continue to age in China, the effectiveness and cost-efficacy of preventive health services, especially physical examinations, have become increasingly important in addressing health challenges in an aging society. Previous research on physical examinations has found inconsistent findings on healthcare utilization and expenditures. Therefore, this study aimed to examine the association between preventive physical examinations and healthcare utilization and expenditures among middle-aged and older adults in China. We used data from the China Health and Retirement Longitudinal Study from 2011 to 2018. The independent variables were whether the individual had a physical examination within two months before the interview for outpatient analyses and one year before the interview for inpatient analyses. Six outcomes were assessed: any outpatient/inpatient care use, log-transformed outpatient/inpatient out-of-pocket expenditures, and log-transformed total outpatient/inpatient expenditures. We employed propensity score weighting and fixed effects regression models, controlling for demographics and diagnosed chronic diseases. Sensitivity analyses and subgroup analyses were also conducted. Physical examinations were associated with a 46.1% higher odds of inpatient care utilization and a 49.7% higher odds of outpatient care utilization. While no significant association with inpatient expenditures was observed, outpatient expenditures increased significantly (out-of-pocket by a factor of 1.86; total by a factor of 1.72). These results showed that preventive physical examinations were significantly associated with increased short-term healthcare utilization and outpatient expenditures. It emphasized the importance of ensuring insurance coverage, especially for vulnerable populations. More research is needed to develop causal pathways and to examine the long-term impacts.

